# Monk parakeets ‘test the waters’ when forming new relationships

**DOI:** 10.1098/rsbl.2025.0399

**Published:** 2025-11-12

**Authors:** Claire L. O'Connell, Gerald G. Carter, Annemarie van der Marel, Elizabeth A. Hobson

**Affiliations:** ^1^ Department of Biological Sciences, University of Cincinnati, Cincinnati, OH, USA; ^2^ Department of Ecology and Evolutionary Biology, Princeton University, Princeton, NJ, USA; ^3^ Smithsonian Tropical Research Institute, Balboa, Ancón, Panama; ^4^ Howard Hughes Medical Institute, Chevy Chase, MD, USA; ^5^ Institute of Ecology and Biodiversity, Santiago, Chile

**Keywords:** parrot, affiliative relationships, pair bonding, social dynamics, animal sociality

## Abstract

Initiating and developing social relationships with strangers can provide fitness benefits, but it is an inherently risky process. To mitigate potential risks and develop trust, strangers may ‘test the waters’ by gradually escalating the type of social investment from low-cost to high-cost. Opportunities to capture the moment animals first encounter one another in the wild are rare, and detailed quantitative assessments of when and how animals initiate relationships are limited. We introduced four unfamiliar groups of feral monk parakeets together into a single 22-bird group and observed the sequence of social behaviours that occurred as relationships developed over 22 days. We tested the effect of relationship status (stranger versus familiar) on the probability of dyads following predicted sequences and whether strangers who progressed their relationships maintained higher rates of no-contact proximity compared with dyads that did not. We found that stranger dyads, but not familiar dyads, were more likely to (i) approach each other without contact before making contact and (ii) follow predicted sequences of affiliative behaviours. Strangers that progressed to contact also had higher rates of associations than did birds that never made contact. These findings provide support for ‘Testing the Waters’ during new relationship formation in a socially and cognitively complex species.

## 1. Introduction

Initiating and developing affiliative relationships with unfamiliar (stranger) conspecifics is an important prerequisite for strong social bonds that yield fitness benefits [1–3]. However, building new relationships with strangers is an inherently risky process. Cooperative investments of time and energy can be taxing and costly when a partner defects. Similarly, affiliative behaviours can be risky if unwanted or hasty attempts to make contact result in aggression or injury [4,5].

‘*Raising the Stakes*’ is a game-theoretic strategy for reducing the risks of making cooperative investments in new partners that might not fully reciprocate; it involves starting with minimal investments and escalating the amount of investments only if the partner matches the effort [6]. This strategy was supported by economic cooperation games played by anonymous humans [7] and in the establishment of food-sharing relationships in common vampire bats (*Desmodus rotundus*). In the latter case, previously unfamiliar female vampire bats established lower cost social-grooming relationships before escalating to costlier food-sharing relationships, presumably to assess the reliability of cooperation partners [8]. However, the cooperation strategy *Raising the Stakes* might be an example of a more general strategy to reduce social uncertainty when strangers first meet [8,9], which would apply to social interactions beyond cooperation. At the earliest stages of relationship formation, animals might sample the behaviour of unfamiliar individuals by escalating from lower risk behaviours (e.g. approaching) to higher risk behaviours (e.g. touching). We call this process of transitioning to different higher risk behaviours ‘*Testing the Waters*’ to distinguish it from escalating the amount of costly investments in *Raising the Stakes. Testing the Waters* might be important for establishing preferred partners, but it does not require reciprocal investments.

**Figure 1.**
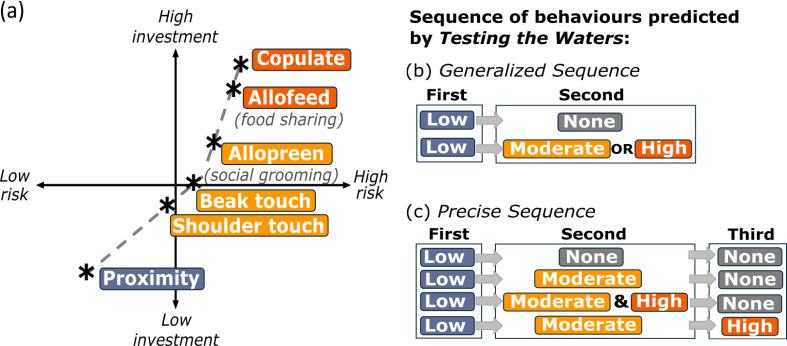
A conceptual diagram of relative risk and investment of behaviours and predicted sequences. (a) The perceptions of relative risk and investment of behaviours with colour indicating the category of behaviour as low, moderate or high. The predicted sequence under *Testing the Waters*: (b) the generalized sequence, which includes dyads that were observed using a low (proximity) behaviour before any type of moderate or high behaviours (affiliative contact) and (c) the precise sequence that includes dyads that were observed using low behaviour before moderate then high, or moderate and high simultaneously (i.e. within the same time bin).

Testing how new relationships form is challenging because opportunities to capture the moment animals first encounter one another in the wild are rare, and detailed quantitative assessments of how animals initiate relationships are even more limited. There has yet to be an explicit assessment of *Testing the Waters* in non-human animals outside of food-sharing vampire bats [8], and a critical question is whether it is generalizable to other social animals and behaviours. If *Testing the Waters* is important during relationship formation, then an important initial behaviour should be relatively low-cost investments like approaches that cause spatial proximity without contact (figure 1). Approaching potential partners does not require large investments of time or energy; it gives individuals time to flee if necessary and it provides the opportunity to observe or signal intent [10–14]. Moderately costly investments that require physical contacts, like social grooming, pose risks like disease transmission [15], aggression [16] and potentially longer term social repercussions (e.g. pile-on aggression, loser-effects) [17,18], while directing time away from self-directed behaviours or vigilance [19] (figure 1a). Finally, high-cost investment contacts that entail nutritional investments (food sharing) or gametic investments (copulation) in partners require the largest investments and should occur only after potential partners are thoroughly vetted [8,20–23] (figure 1a). Therefore, *Testing the Waters* predicts that animals should approach and maintain no-contact proximity on multiple occasions (or for prolonged periods) before touching becomes common.

Here, we investigated the sequences of behaviours that occurred during relationship formation in monk parakeets (*Myiopsitta monachus*), which are small Neotropical parrots that exhibit social and cognitive complexity [24–28]. Wild and captive birds readily form mixed flocks with familiar and unfamiliar birds, and although they maintain clear preferences for familiar social partners, they can also form affiliative relationships with strangers within a few days [29]. Birds selectively form strong affiliative relationships with a few same- or mixed-sex social partners in the wild and in captivity [24,25,29–32]. Because of these documented patterns, we expected that the sequence of behaviours predicted by *Testing the Waters* should occur in all new dyads regardless of the sex composition of the dyad or their motivations for escalating their relationship.

To determine how affiliative relationships develop, we introduced feral groups of monk parakeets in a captive setting and tracked the emergence of affiliative interactions. We tested (i) whether the probability of observing the sequence of behaviours predicted from *Testing the Waters* [8] occurred more often among strangers than among familiar birds and (ii) whether the probability of observing the predicted sequence among strangers was greater than the probability expected from random interactions. We also tested (iii) whether strangers who progressed their relationships maintained higher rates of no-contact proximity compared with stranger dyads that did not. We show that stranger dyads, but not familiar dyads, progressed according to *Testing the Waters*. Finally, we show that strangers that progressed to contact also had higher rates of associations than did birds that never made contact.

## 2. Methods

### (a) Social group and experimental design

We used feral monk parakeets (*n* = 22; eight females and 14 males) captured just prior to the experiment from four geographically distinct sites (site 1: *n* = 5, site 2: *n* = 6, site 3: *n* = 7 and site 4: *n* = 4). Birds from different capture sites were very likely unfamiliar to one another prior to the start of the experiment due to the distances between their capture locations (mean: 16.06 km; range: 3.28–30.85 km) [29]. All newly captured birds underwent a quarantine period that allowed them time to acclimate to their new diets but was short enough that the birds were still feral (approx. 3–6 weeks). During this time, we maintained stranger status by only housing birds together if they were caught at the same site and blocking visual contact between birds from different populations, ensuring that birds from different populations never saw each other before the experiment. All birds were in vocal contact. We randomly assigned birds a unique three-colour combination to readily identify individuals from multiple angles during the experiment. Marks were applied to each bird’s head, cheek and chest with non-toxic permanent markers (Sharpie^®^ Inc.) several days before the experiment began [24,25,29,30] and were refreshed immediately before birds were introduced into the flight pen.

The experiment occurred for 22 days over 132.3 observation hours in April 2021 at the United States Department of Agriculture, Wildlife Services, National Wildlife Research Center, Florida Field Station, in Gainesville, FL, USA, and took place in a 2025 m² semi-natural outdoor flight pen constructed entirely from metal mesh. Birds had constant access to a seed mix, water, grasses, trees and artificial perches within the flight pen (see flight pen conditions detailed in O’Connell *et al.* [29]). A team of four observers recorded interactions from blinds in three locations, providing different viewing angles of the flight pen (see map in Estien *et al.* [33]). Observers recorded spatial associations and dyadic interactions using an all-occurrence sampling method [34] with the Animal Observer application on iPads [35–37].

To initiate the experiment, all 22 birds were released simultaneously into the flight pen. Observers began collecting proximity and social data immediately on release. Four observers simultaneously collected data. Daily observation sessions took place between 08.00 and 19.00 and were typically split into morning and evening sessions to capture periods of the day where birds’ activity was the highest. Observers took breaks in shifts such that at minimum of two observers were always present.

### (b) Social behaviours and predicted sequences

To quantify approaches and proximity in the absence of touching, observers recorded a bird’s *nearest neighbour* that was peacefully present without physical contact within a maximum distance of 1 m, at a rate of once every 5 min. Observers recorded five affiliative physical interactions as they occurred: (i) s*houlder contact* (two birds perched next to each other so that they are touching), (ii) *allopreening* (social grooming), (iii) *beak touching* (birds simultaneously touching beaks), (iv) *allofeeding* (the regurgitation of food into another’s beak) and (v) *copulation*.

We performed all data analyses in R v. 4.3.2 [38]. We analysed observations from confidently identified individuals. To eliminate duplicate entries (e.g. when the same interaction was recorded by more than one observer during data collection) and to account for frequency differences in continuous behaviours, we coded each interaction as either occurred (1) or absent (0) for each dyad in each 5-min time interval (timebin).

We categorized the first observed instances of behaviours based on perceptions of relative risk and investment into three levels (figure 1a): low (no-contact proximity), moderate (shoulder contact, allopreening and beak touching) or high (allofeeding and copulating), based on past work in monk parakeets [29] and other species [8,39–41]. These categories captured levels of relationship progression based on predicted patterns of social investment while allowing for some variability in how relationships progress. After we identified the first observation of each behaviour within dyads, we determined whether the sequence of the behaviours progressed according to a generalized sequence and a precise sequence, predicted by *Testing the Waters* (figure 1b,c). Compared with the generalized sequence, the precise sequence provided a more detailed evaluation of relationship progression. The observed sequence matched the generalized sequence if a dyad progressed through low before any type of moderate or high behaviours (figure 1b). The observed sequence matched the precise sequence if a dyad progressed through low behaviours before moderate then high, or moderate and high simultaneously (figure 1c). However, if the first observation of a low or moderate behaviour followed the first observation of a high behaviour, then this observed sequence contradicted our predicted order. Therefore, each dyad was categorized as having supported *Testing the Waters* or not (true/false).

### (c) Estimating the effect of relationship status on the sequence of behaviours

To assess whether the predicted sequences occurred more often in stranger dyads than in familiar dyads, we used the R package brms (v. 2.220) [42–44] to fit two generalized multi-membership models with a Bernoulli outcome (logit link) for whether or not dyads followed the expected sequence. In the first model, the expected sequence was the generalized sequence and in the second model, it was the precise sequence. Multi-membership models use group-structured random effects to control for the dependence of each dyad on the identities of both individuals [45]. The fixed effect of interest was whether the dyad was familiar or not. To test for potential sex effects, we also tested a model including the dyad’s combination of sex as a fixed effect (MF, MM, FF; additional methods in electronic supplementary material,1.2). We ran four Markov chain Monte Carlo (MCMC) chains with 8000 samples and a warm-up period of 4000. We assessed MCMC convergence by examining trace and posterior predictive plots and by ensuring Rhat values = 1.00 (electronic supplementary material, 2.1 and 2.2). We used default uninformative priors. If *Testing the Waters* shapes new relationship formation, we predicted that stranger dyads would have a greater probability of performing the predicted sequences compared with familiar dyads.

To simulate how often predicted sequences would occur if stranger and familiar dyads had exhibited behaviours in random order, we shuffled (1000 iterations) the time bin in which behaviours were observed among dyads. This randomization controlled for the relative frequency of behaviours, because some interactions were more likely to occur first simply because they were more frequent. We then estimated the probability that dyads matched the generalized or precise sequences given random ordering (figure 1b,c).

### (d) Testing for relationship progression among strangers

Using the same permutation-based reference model described above, we also used non-parametric permutation tests to assess whether the sequences occurred more than expected if stranger dyads had exhibited behaviours in a random order. In the generalized sequence test, we tested whether no-contact proximity preceded any type of affiliative contact between stranger dyads (figure 1b). To do this, we compared the probability of observing low behaviours before any type of moderate or high behaviours with the probabilities from random expectations. In the precise sequence test, we tested whether proximity preceded a more defined sequence of affiliative interactions between stranger dyads (figure 1c). To do this, we compared the observed probability of observing low behaviours before moderate then high, or moderate and high simultaneously. We used one-sided *p*-values because our hypothesis was unidirectional.

Finally, we assessed whether each bird’s mean hourly proximity rate with stranger dyads observed in affiliative contact was greater than the mean hourly proximity rate with stranger dyads not observed in affiliative contact, using a permuted paired *t*‐test (5000 iterations). We only averaged hourly proximity rates prior to each dyad’s first contact. We expected that mean hourly proximity rates would be greater among dyads observed in contact, as expected if these dyads first approach and maintain proximity with preferred partners before contact.

## 3. Results

**Figure 2.**
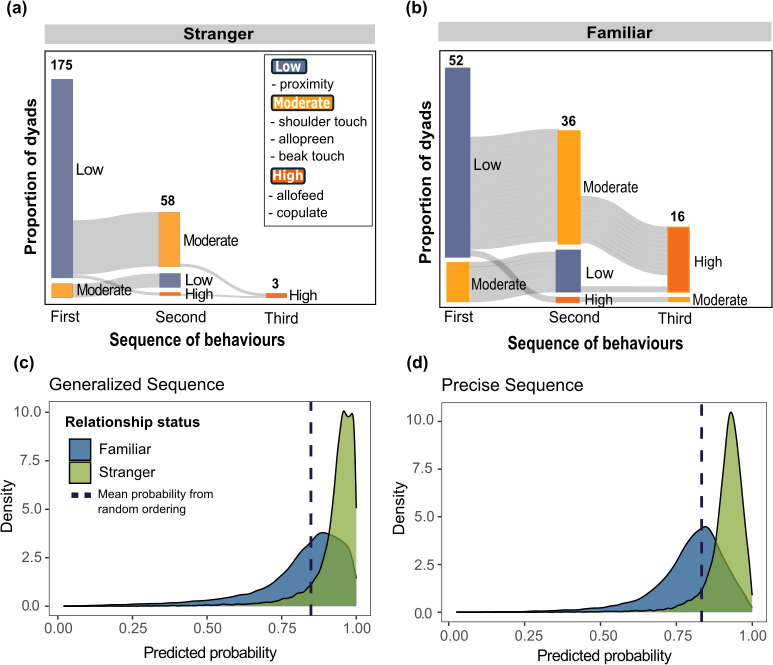
The predicted sequences were more common among stranger dyads compared with familiar dyads**.** The proportion of (a) stranger dyads and (b) familiar dyads progressing (grey) from each behaviour, visualized with the R package Sankey (v. 0.4) [46]. The height of the bar indicates the proportion of total dyads that were observed interacting. The label above the bars shows the total number of dyads observed in the sequence. The colour of the bars represents the estimated category of relative risk and investment of the behaviour (see figure 1). The posterior probability distributions for the probability of observing the (c) generalized and (d) precise sequence among stranger dyads (green) and familiar dyads (blue), estimated from the Bayesian multi-membership model. The probabilities expected from random orderings are shown by vertical dashed lines.

We observed diminishing frequencies for social behaviours categorized as ‘low’, ‘moderate’ and ‘high’ based on their relative risk and investment. Of the 179 initial stranger dyads, we observed 175 dyads (98%) using low, 56 dyads (31%) using moderate and five dyads (2.9%) using high behaviours. Of the 52 familiar dyads, we observed 100% using low, 36 dyads (69%) using moderate and 16 dyads (31%) using high behaviours. To see summaries of relationships that progressed see figure 2a,b, we found no effect of sex composition of the dyad on the predicted sequences (see electronic supplementary material, 1.1).

**Figure 3.**
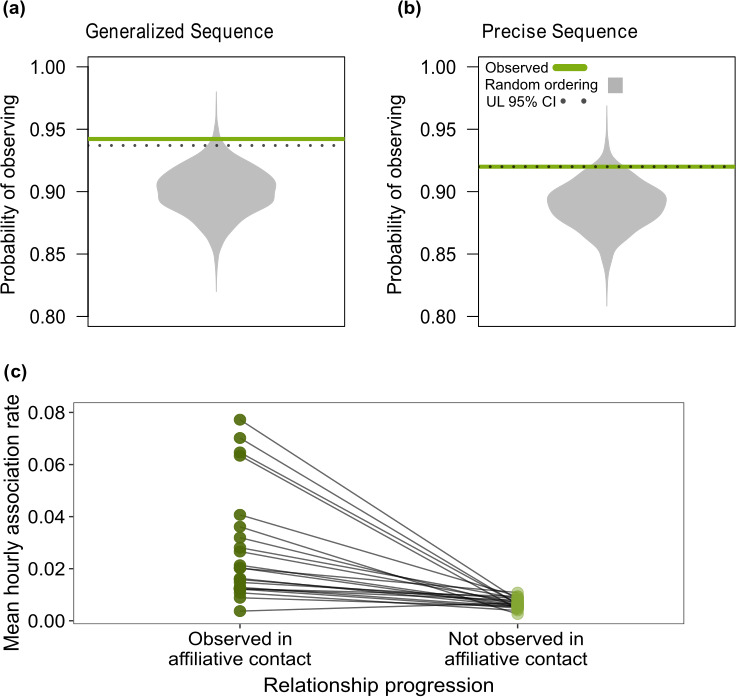
Stranger dyads progressed according to predicted sequences. Comparisons of the observed probabilities (green) of observing predicted (a) generalized sequence (figure 1b) and (b) precise sequence (figure 1c) across behaviours to the probabilities expected from random ordering (grey distributions), visualized with R package Beanplot [47]. The upper 95% quantile for expected values is indicated by dotted lines. (c) Each birds’ mean hourly association rates that occurred prior to affiliative contact with stranger dyads they were observed in affiliative contact with and stranger that were never observed in affiliative contact.

We found a strong difference in how often stranger and familiar birds followed sequences expected from *Testing the Waters* (figure 2). The predicted sequences were more common among stranger dyads than familiar dyads, which was consistent with our prediction. Specifically, being a stranger dyad changed the odds of following the generalized sequence by a factor of 3.39 (log odds = 1.22, 95% Bayesian credible interval (CI) = [0.16 – 2.27]; electronic supplementary material, 3) and the precise sequence by an estimated factor of 2.89 (log odds = 1.05, 95% CI = [0.12 – 1.98]; electronic supplementary material, 3). The mean probability of the predicted sequence was greater than expected from random ordering, but only for stranger dyads, and not for familiar dyads (generalized sequence: random expectation = 85%; stranger dyad probability = 0.95, [0.90, 0.98]; familiar dyad = 0.86 [0.70, 0.95], figure 2c; precise sequence: random expectation = 83%, stranger dyad = 0.93 [0.87, 0.96], familiar dyad = 0.81 [0.68, 0.91], figure 2d). Our more conservative non-parametric permutation tests further confirmed that the probability of observing the generalized sequence in stranger dyads was significantly greater than expected from randomized behavioural sequences (observed = 0.94; *p*‐value = 0.01; figure 3a). The probability of observing the precise sequence was nearly statistically significant (obs. = 0.92; *p*‐value = 0.06; figure 3b). Finally, as expected from *Testing the Waters*, for each bird, mean hourly association rates were greater between stranger dyads that progressed to contact compared with stranger dyads without contact (*n* = 22; with contact: 0.03 ± 0.02; without contact: 0.01 ± 0.00; *p*‐value < 0.0001; figure 3c).

## 4. Discussion

We introduced groups of unfamiliar monk parakeets in a captive setting and observed the sequence of first-occurrence behaviours as new social relationships developed. We found that the sequences predicted by *Testing the Waters* were more common in stranger dyads than in familiar dyads and were more common than expected from a random ordering of behaviours. Strangers that progressed to contact also had higher rates of associations than did birds that never made contact. This study is, to our knowledge, the first compelling support for *Testing the Waters* in a species beyond vampire bats [8] and across more than two social behaviours.

Our results suggest that maintaining spatial proximity to gain familiarity or to minimize social uncertainty before physical contact was a critical initial stage in developing new affiliative relationships with preferred partners. We found that birds more often approached the strangers that they later physically contacted compared to strangers that they were never observed to contact. This difference in associations was unlikely to be due to strangers simply avoiding each other. In previous work with this study population, we showed that although strangers did not immediately affiliatively interact, they readily shared space and showed no evidence of avoiding one another [29,48]. Together, these findings indicate that no-contact proximity before affiliative contact was a decision made by strangers, not a by-product of the birds’ spatial assortment or the frequencies of different behaviours.

We found strong evidence for *Testing the Waters* in new relationships among stranger dyads but not familiar dyads. The high percentages of moderate and high behaviours and the lack of predicted patterns between familiar dyads suggest that these relationships developed and stabilized prior to the experiment. Generally, stable social bonds should persist across social and physical contexts. For example, female vampire bats that developed or strengthened allogrooming and allofeeding relationships in captivity maintained the same relationships following their release into the wild [49]. In our study, a lack of evidence for *Testing the Waters* among familiar dyads suggests that these relationships remained intact following their introduction into the flight pen, while evidence for *Testing the Waters* among stranger dyads suggests this process was necessary to develop new relationships. Because we did not observe the familiar birds’ relationships in the wild prior to their capture, we cannot know whether they had pre-existing affiliative relationships or were simply familiar. Therefore, it remains unclear whether *Testing the Waters* would be used only with strangers or would be used with any individual prior to any affiliative interaction occuring.

We did not find that sex composition of familiar or stranger dyad predicted the sequence of behaviours, despite experiments taking place during peak breeding season for populations of monk parakeets in South Florida [32], suggesting that this process is important regardless of motivations (reproductive or affiliative) for escalating relationships.

Although we found strong support for this social group of monk parakeets’ capacity for *Testing the Waters* in captivity, studies in the wild are necessary to determine whether this pattern is also followed in wild populations. Throughout their native and expanded range, monk parakeets live in large social groups and often build communal stick nests with other breeding pairs that temporarily merge with neighbouring social groups to forage [24,25,31,32]. Forming these relationships appears to be central to their socioecology [24] and is thought to play a critical role in their success in establishing breeding populations outside of their native range [32]. We expect similar patterns in wild populations to what we observed given that our subjects were newly caught and the experiment took place in a naturalistic outdoor setting.

Thus far, *Testing the Waters* has only been formally investigated to explain patterns of affiliative relationship development or how reciprocal cooperation emerges [6,8], but it could also be important for establishing relationships in other behavioural contexts including preventing aggression. For example, in male Verreaux’s sifaka lemurs (*Propithecus verreaux*) play was more common among strangers from visiting social groups compared with familiar residents. After play, rates of aggression towards strangers decreased, which is consistent with the authors’ suggestion that play acts as an ‘ice-breaker’ to establish trust and reduce social uncertainty [9]. Such cross-over effects between behavioural contexts may occur across other species and affect novel relationship formation among strangers. Because rank and aggression are important features of monk parakeet social groups [22–24,26,28], *Testing the Waters* could be used by the birds to reduce the risk of receiving aggression or losing rank in a dominance hierarchy.

*Testing the Waters* may be a widespread mechanism for relationship formation for social species [50–52]. Further cross-species comparisons with wild and captive social groups are necessary to gain insight into the mechanisms of formation and how animals balance the risks and benefits of associating in the presence of social uncertainty.

## Data Availability

Publishable data accessibility statement: data and R code are available on Dryad: [53]. Supplementary material is available online [54].
